# Supplementary material for: Fermi liquid-like behaviour of cuprates in the pseudogap phase simulated via T-dependent electron-boson spectral density
Source: Sci Rep. 2023 Feb 13;13:2527. doi: 10.1038/s41598-023-29829-w (PMC9925830; doi:10.1038/s41598-023-29829-w)
Supplement: Supplementary file 1 — Supplementary Figures. [file 41598_2023_29829_MOESM1_ESM.pdf]

Supplementary Material

Fermi liquid-like behaviour of cuprates in the pseudogap phase simulated via *T*-dependent electron-boson spectral density

Hwiwoo Park and Jungseek Hwang*

Department of Physics, Sungkyunkwan University, Suwon, Gyeonggi-do 16419, Republic of Korea

Temperature-dependent changes of the parameters in the EBSD function

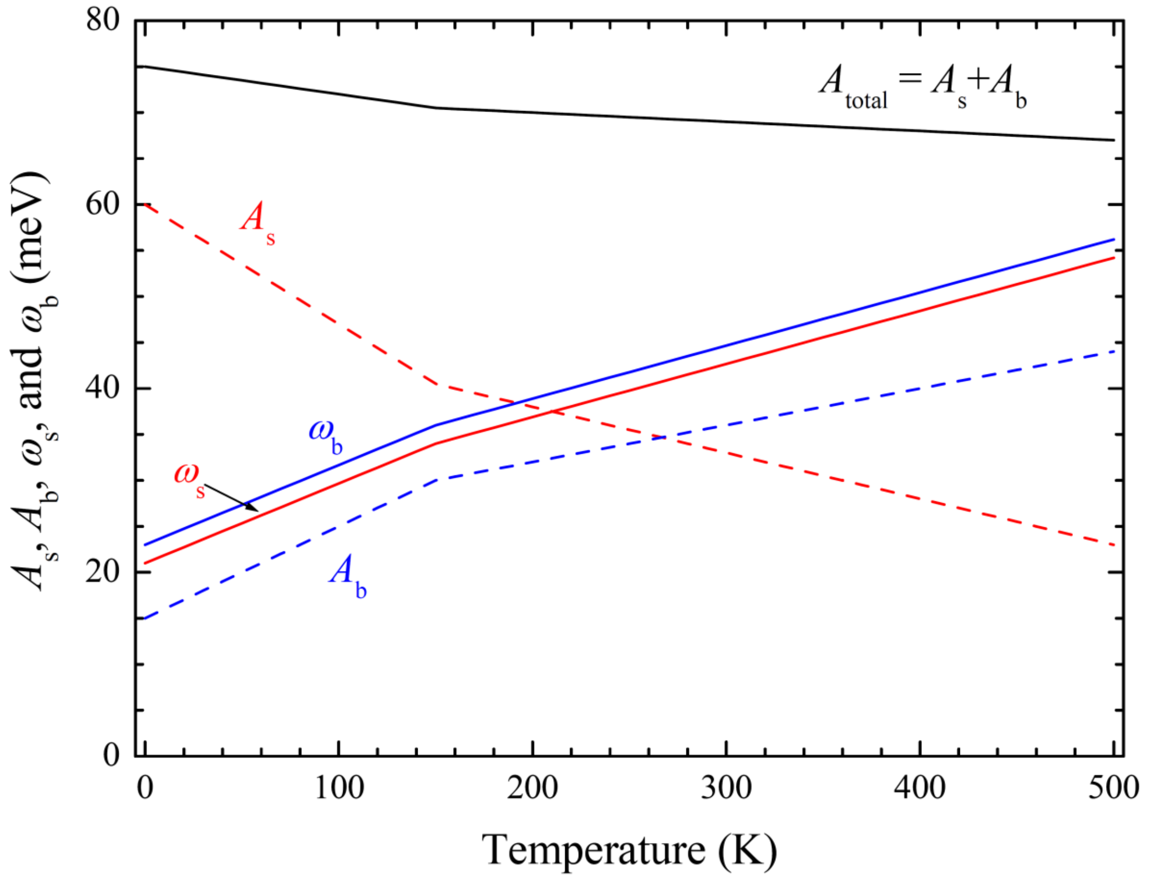

Figure S1: Temperature-dependent changes of the parameters in the *T*-dependent EBSD function (see Eq. (4) in the main text).

Total optical self-energy including the impurity scattering rate for the case of PG

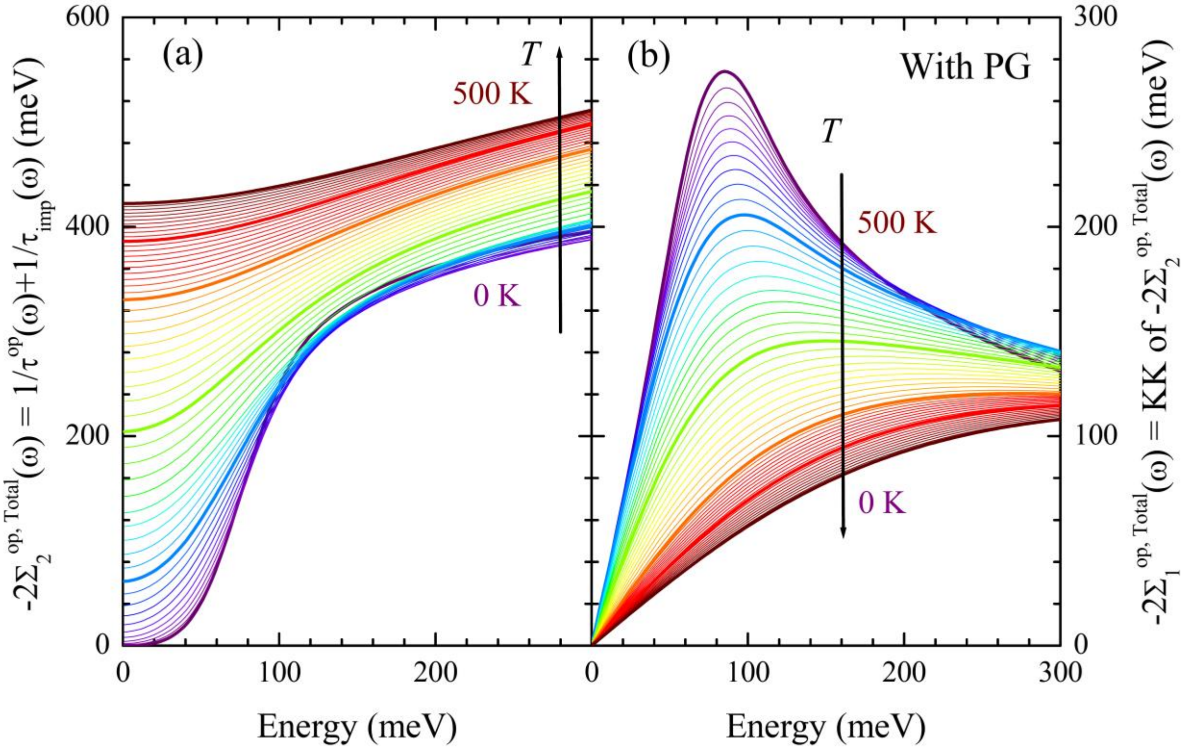

Figure S2. (a) Resulting optical scattering rate including the impurity scattering rate ($-2\Sigma_2^{op,\mathrm{Total}}(\omega)$) and (b) the corresponding real part of the optical self-energy ($-2\Sigma_1^{op,\mathrm{Total}}(\omega)$) obtained from the resulting optical scattering rate using the Kramers-Kronig (KK) relation at various temperatures ranging from 0 to 500 K with a 10 K increment. These are used to obtain the optical conductivity shown in Fig. 6 in the main text. We note that the thick purple is 0 K, the thick blue curve is 100 K, the thick green curve is 200 K, the thick orange curve is 300 K, the thick red curve is 400 K, and the thick dark brown curve is 500 K.

Drude-Lorentz model fit of the optical conductivity in the case without including PG

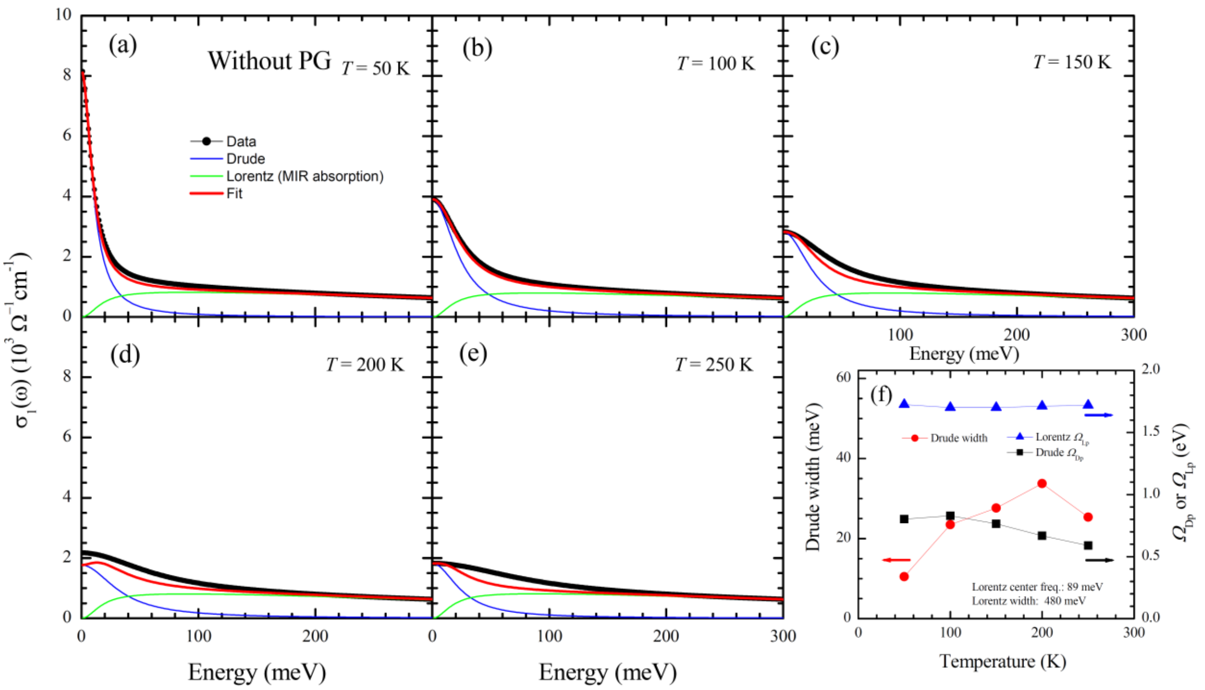

Figure S3. (a-e) The optical conductivity data and Drude-Lorentz model fits at five selected temperatures in the case without including the PG. The data were fitted up to 300 meV with a Drude mode and a Lorentz mode. (f) The temperature-dependent fitting parameters are the Drude plasma frequency (Ω_{Dp}) and width, and the Lorentz center frequency, width, and plasma frequency (Ω_{Lp}). Note that the center frequency and width of the Lorentz mode are fixed. The Lorentz mode is almost T-independent,whereas the Drude mode shows strong T-dependence.

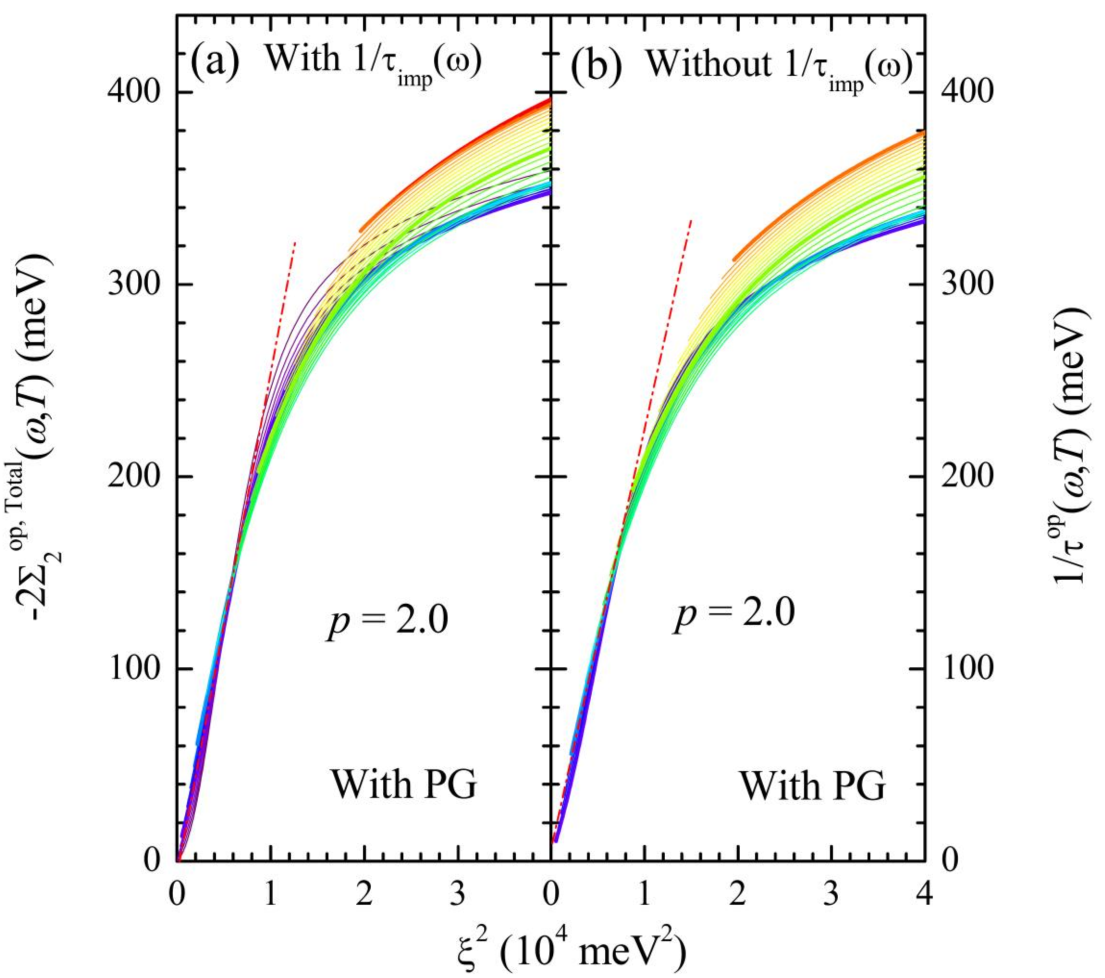

Figure S4. For T-dependent EBSD function, (a) Total optical scattering rates ($-2\Sigma_2^{op,\mathrm{Total}}(\omega)$) including the impurity scattering rates as functions of $\xi^2 \equiv (\hbar\omega)^2 + (p\pi k_B T)^2$ for p = 2.0 include PG. (b) Optical scattering rates ($(1/\tau^{op}(\omega))$) as functions of $\xi^2 \equiv (\hbar\omega)^2 + (p\pi k_B T)^2$ for p = 2.0 include PG, which is identical with Fig. 6(b) in the main text. The two sets of optical scattering rates exhibit similar behaviors. Here, the optical scattering rates at temperatures between 50 and 300 K are displayed, including only the pesudogaps with reasonable pesudogap depths. The dash-dotted lines are guides for the eyes.

Results from temperature-independent electron-boson spectral density (EBSD) function

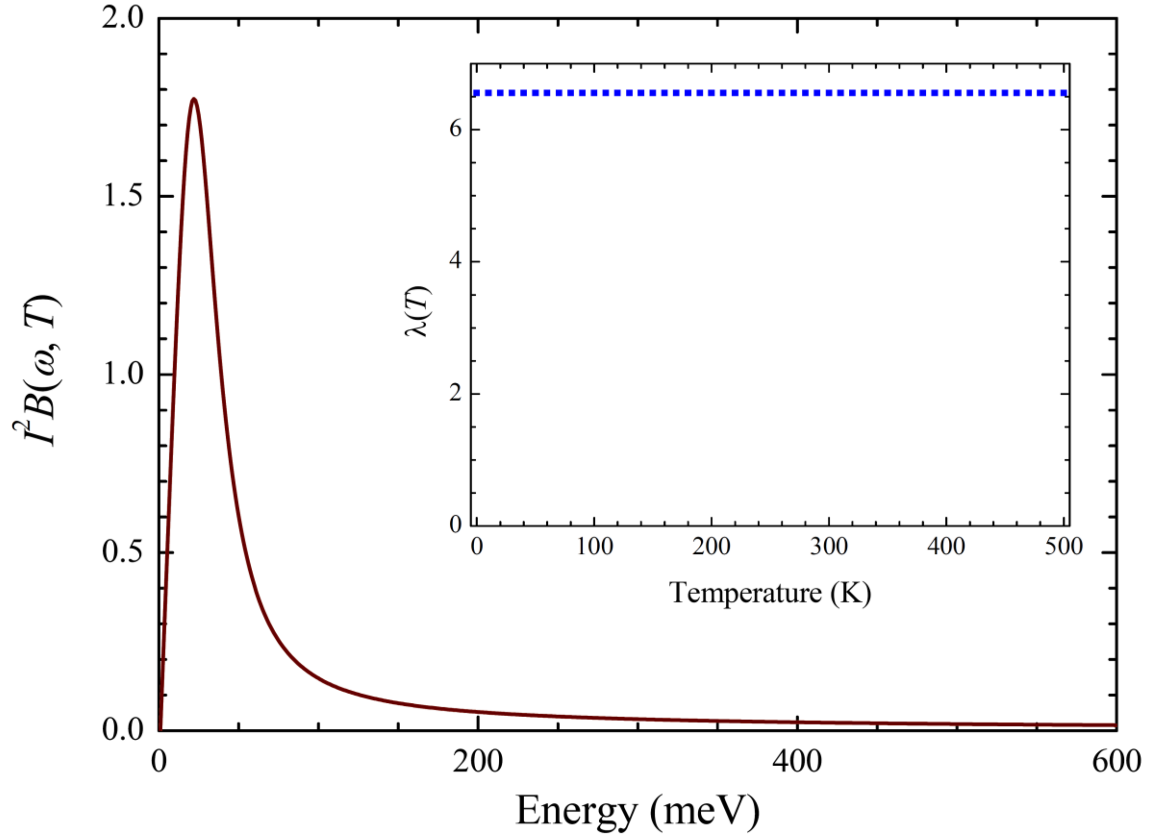

Figure S5. Electron-boson spectral density (EBSD) function, which consists of two (sharp and broad) components. In the inset, the temperature-independent coupling constant ($\lambda(T)$) is shown. The EBSD is temperature independent and consists of two components as described in Eq. (4) in the main text. The parameters are as follows: A_b = 15 meV, ω_b = 23 meV, A_s = 60 meV, $\omega_s/\sqrt[4]{3}$ = 21 meV, and ω_c = 625 meV.

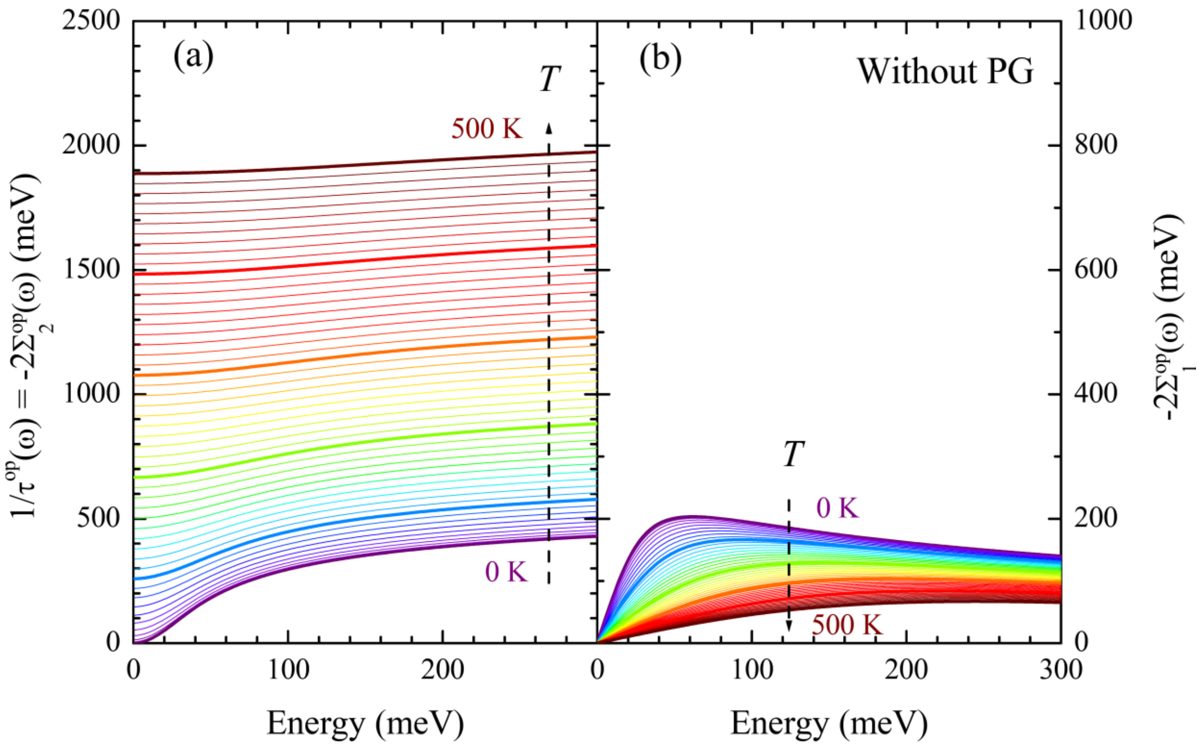

Figure S6. (a) Optical scattering rates (or imaginary parts of the optical self-energy) and (b) corresponding real parts of the optical self-energy at various temperatures ranging from 0 to 500 K with a 10 K increment without including the pseudogap. The real parts of the optical self-energy were obtained from the imaginary parts using the Kramers-Kronig relation between the real and imaginary parts.

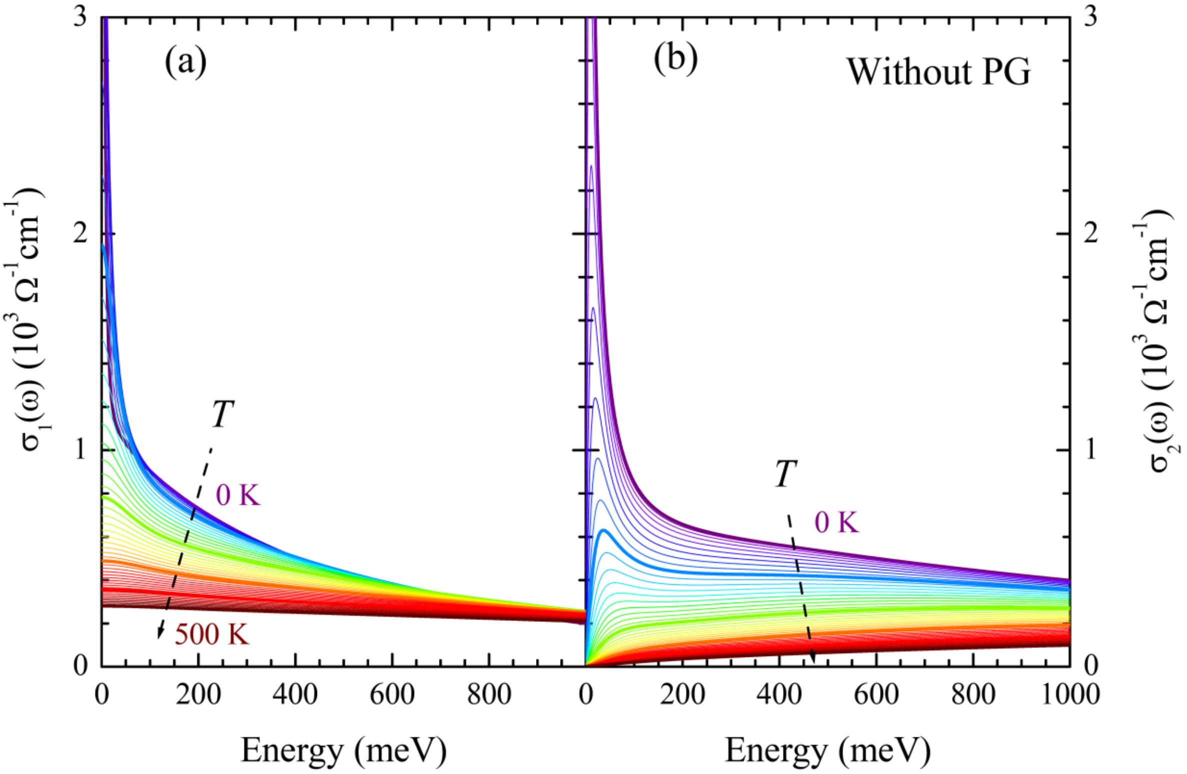

Figure S7. (a) Real and (b) imaginary parts of the optical conductivity at various temperatures between 0 and 500 K. The complex optical conductivities were obtained from the complex optical self-energy using the extended Drude model, Eq. (1) in the main text, for the case of the *T*-independent EBSD function without including the pseudogap. The plasma frequency (Ω_p) is 2.0 eV and the impurity scattering rate ($1/\tau_{imp}$) is 15 meV.

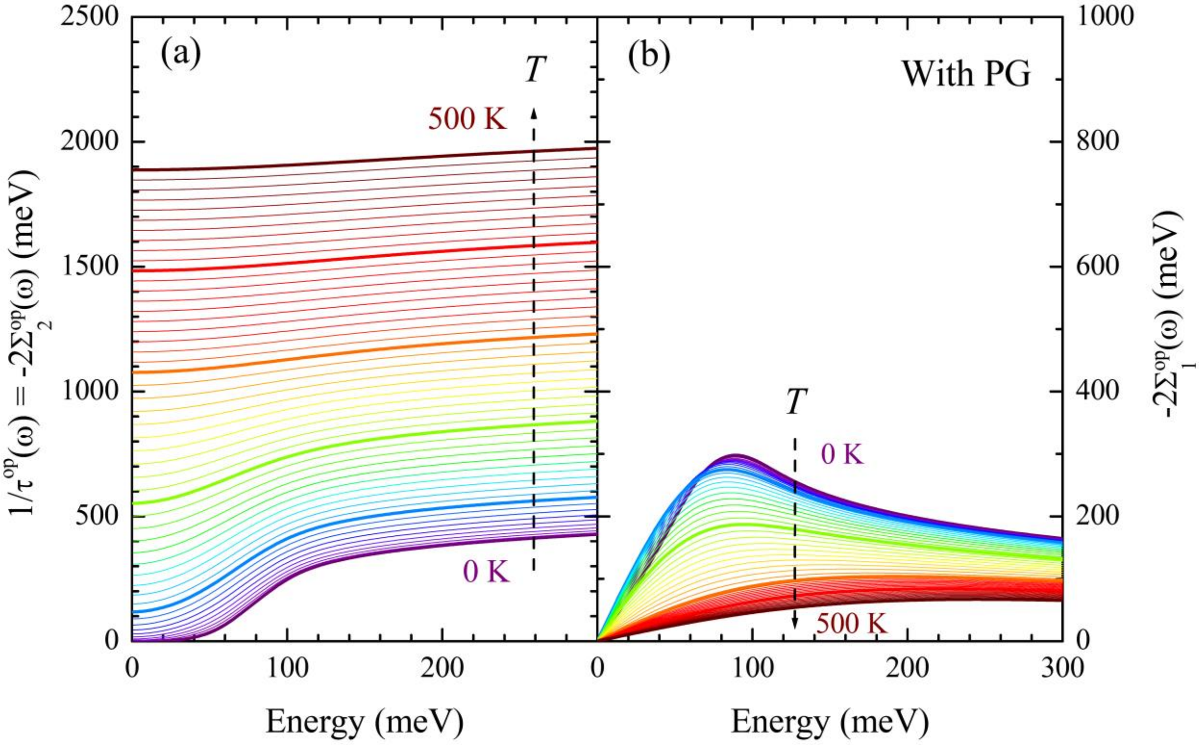

Figure S8. (a) Optical scattering rates (or imaginary parts of the optical self-energy) and (b) the corresponding real parts of the optical self-energy at various temperatures ranging from 0 to 500 K with a 10 K increment including the pseudogap. The same temperature-dependent pseudogap model in the main text (see the inset of Fig. 4) is used for the pseudogap.

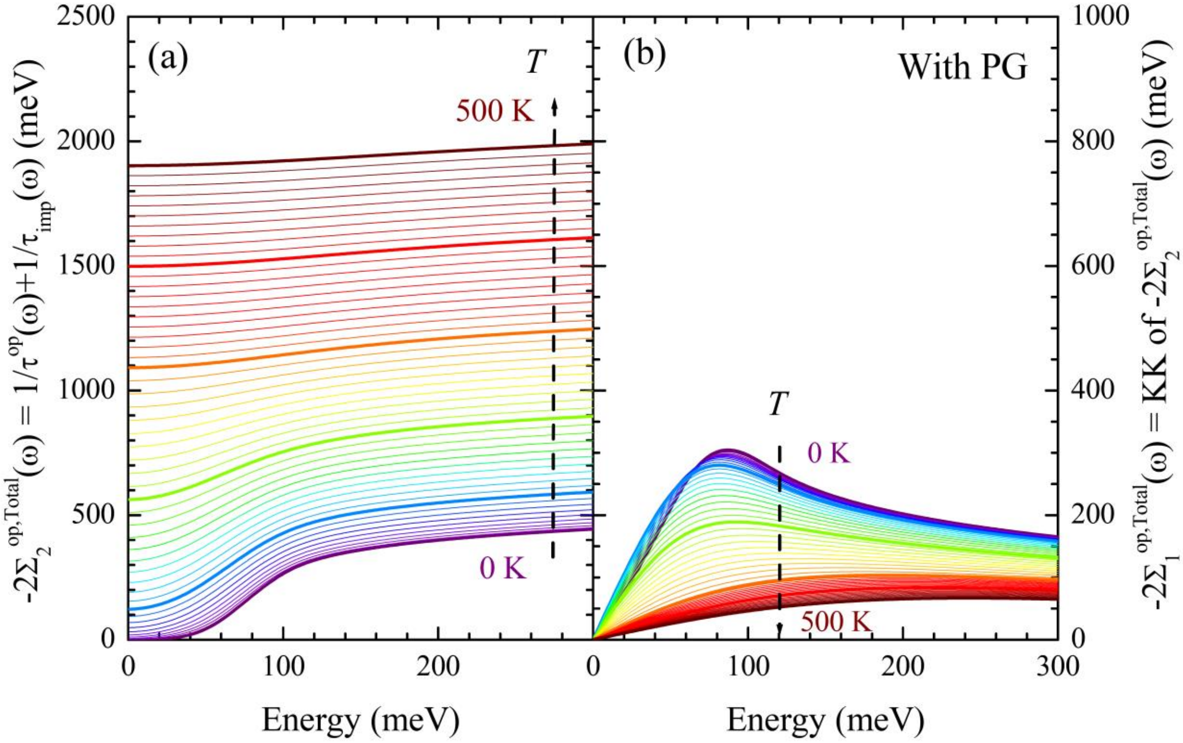

Figure S9. (a) Total optical scattering rate including the impurity scattering rate ($-2\Sigma_2^{op,\mathrm{Total}}(\omega)$) and (b) the corresponding real part of the optical self-energy ($-2\Sigma_1^{op,\mathrm{Total}}(\omega)$) obtained from the resulting optical scattering rate using the Kramers-Kronig (KK) relation. These are used to obtain the optical conductivity shown in Fig. S8.

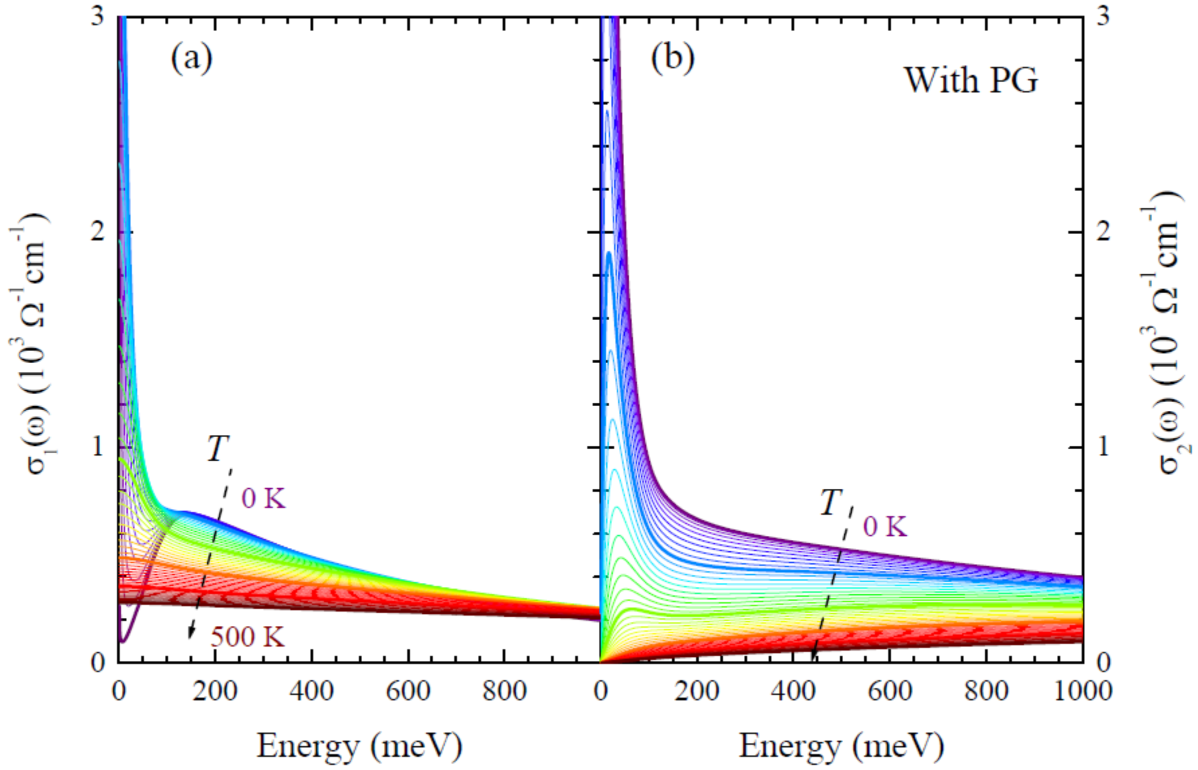

Figure S10. (a) Real and (b) imaginary parts of the optical conductivity at various temperatures between 0 and 500 K. The complex optical conductivities were obtained from the complex optical self-energy using the extended Drude model, Eq. (1) in the main text, for the case of the T-independent EBSD function including the pseudogap. The plasma frequency (Ω_p) is 2.0 eV, and the frequency-dependent impurity scattering rate ($1/\tau_{imp}(\omega)$) with 15 meV is used (see the inset of Fig. 5(a) in the main text).

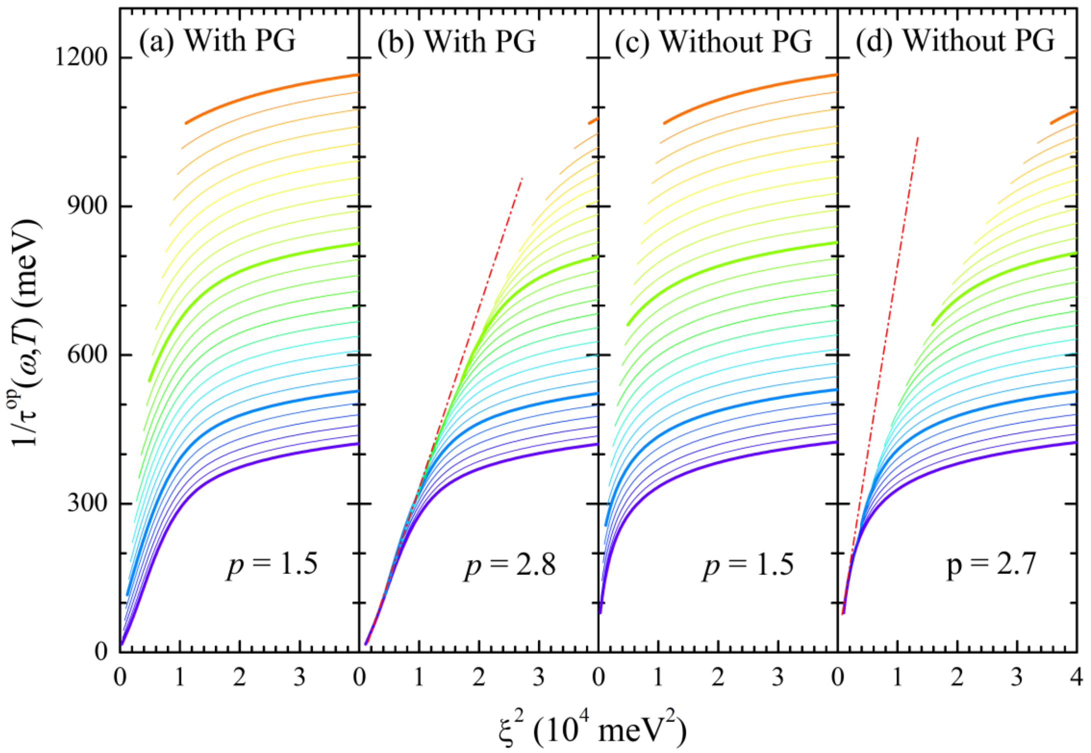

Figure S11: Optical scattering rates as functions of $\xi^2 \equiv (\hbar\omega)^2 + (p\pi k_B T)^2$ for (a) p = 1.5 and (b) p = 2.8 including the PG and (c) p = 1.5 and (b) p = 2.7 without including the PG. Here, the optical scattering rates at temperatures between 50 and 300 K are displayed, including only the pesudogaps with reasonable pesudogap depths. Note that the thick blue curve is 100 K, the thick green curve is 200 K, and the thick orange curve is 300 K. The dash-dotted lines in (b) and (d) are guides for the eyes.

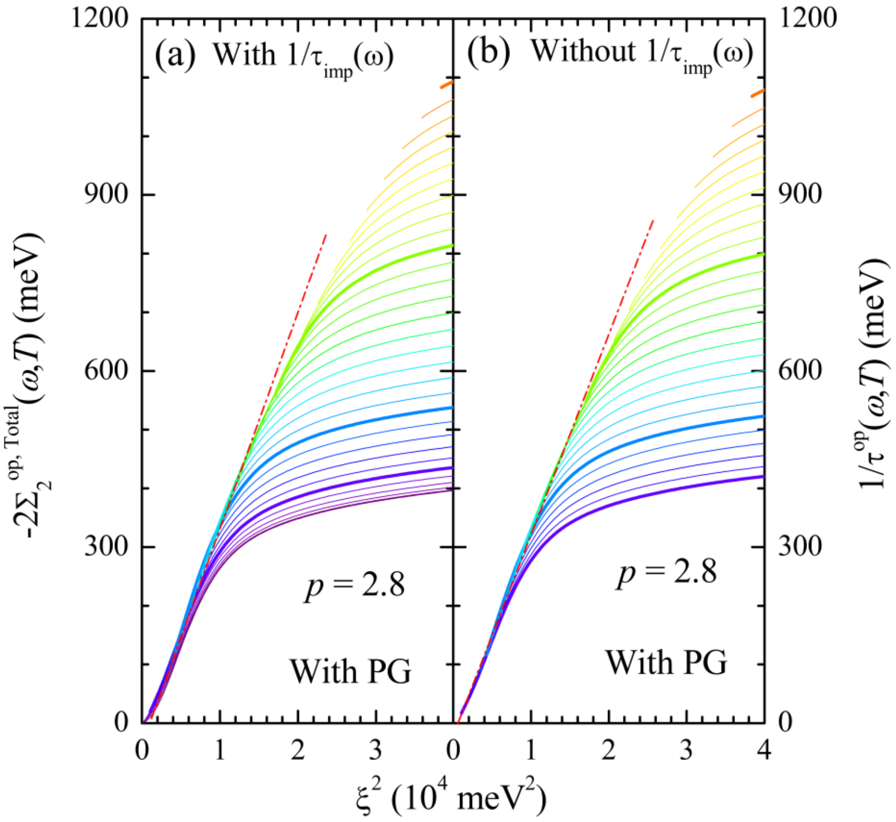

Figure S12. For T-independent EBSD function, (a) Total optical scattering rates including the impurity scattering rates as functions of $\boldsymbol{\xi^2 \equiv (\hbar\omega)^2 + (p\pi k_B T)^2}$ for $p = 2.8$ include PG. (b) Optical scattering rates as functions of $\boldsymbol{\xi^2 \equiv (\hbar\omega)^2 + (p\pi k_B T)^2}$ for $p = 2.8$ include PG, which is identical with Fig. S9(b). The two sets of optical scattering rates exhibit similar behaviors. The dash-dotted lines are guides for the eyes.
